# Job Satisfaction Among Nursing Staff: A Cross-Sectional Study in Slovenian Healthcare Settings

**DOI:** 10.3390/nursrep16040112

**Published:** 2026-03-30

**Authors:** Sebastjan Merlo, Iztok Podbregar

**Affiliations:** 1 Operational Activities Sector, Institute of Oncology Ljubljana, 1000 Ljubljana, Slovenia; smerlo@onko-i.si; 2 Faculty of Medicine, University of Ljubljana, 1000 Ljubljana, Slovenia; 3 Angela Boškin Faculty of Health Care, 4270 Jesenice, Slovenia; 4 Faculty of Organizational Sciences, University of Maribor, 4101 Kranj, Slovenia

**Keywords:** job satisfaction, nursing staff, workload, organizational factors, leadership, job mobility, healthcare workforce

## Abstract

**Background:** Job satisfaction among nursing staff is a key determinant of workforce stability, quality of care, and healthcare system sustainability. Nurses are increasingly exposed to high workload, staffing shortages, and complex organizational demands, which may adversely affect satisfaction and retention. The aim of this study was to examine job satisfaction among nursing staff working across different levels of healthcare in Slovenia and to identify organisational and sociodemographic factors associated with job satisfaction. **Methods:** A cross-sectional quantitative study was conducted among nursing staff employed in Slovenian healthcare settings. Data were collected using an online questionnaire that included the Job Satisfaction Survey (JSS) and sociodemographic, occupational, and organizational variables. Differences in job satisfaction across professional groups were examined using non-parametric tests. Associations between job satisfaction dimensions and explanatory variables were analysed using Spearman’s correlation coefficients, and multiple linear regression analyses were performed to identify independent predictors of job satisfaction. **Results:** Organizational and workload-related factors emerged as the most consistent determinants of job satisfaction across all JSS dimensions and total satisfaction. Unclear job task definitions, high workload, insufficient staffing, continuous healthcare provision, unfavourable work schedules, and limited opportunities for rest were associated with lower job satisfaction. In contrast, financially compensated overtime, supportive supervision, higher perceived employer quality, longer tenure in the current position were associated with higher satisfaction in several domains. Sociodemographic variables showed weaker and less consistent effects after adjustment for organizational characteristics. Intentions to change jobs within or outside the healthcare system were strongly associated with lower satisfaction across nearly all dimensions. **Conclusions:** Job satisfaction among nursing staff is shaped predominantly by modifiable organizational factors rather than demographic characteristics. Interventions aimed at improving task clarity, staffing adequacy, work organization, leadership practices, and recovery opportunities may enhance job satisfaction and contribute to a more sustainable nursing workforce.

## 1. Introduction

Job satisfaction among nursing staff is a key determinant of healthcare workforce sustainability, quality of care, and organisational effectiveness. Contemporary nursing practice is characterised by high workload, staffing shortages, shift work, and complex organisational demands, which have been associated with reduced job satisfaction, burnout, and increased turnover intentions [1,2,3]. Job satisfaction is a multidimensional construct encompassing intrinsic aspects, such as meaningfulness of work, autonomy, and interpersonal relationships, and extrinsic aspects, including remuneration, promotion opportunities, working conditions, and organisational policies [2,4]. While nurses often report higher satisfaction with intrinsic dimensions, dissatisfaction is more frequently observed in extrinsic domains related to organisational structure, staffing adequacy, and resource allocation [1,5,6]. Evidence from international studies indicates that job satisfaction is more strongly influenced by organisational and workload-related factors than by sociodemographic characteristics. High job demands, inadequate staffing, unclear roles, limited recovery time, and continuous care provision have been identified as key predictors of dissatisfaction and turnover intentions across diverse care settings [7,8,9,10,11,12]. Recent research further highlights the role of nurse-to-patient ratios, workload intensity, and missed care in shaping nurses’ work experiences and satisfaction [12,13,14]. Conversely, supportive organisational climates, effective leadership, clear communication, and perceived fairness in compensation systems have been associated with higher job satisfaction and improved retention [15,16,17]. Recognition at work and transparent reward structures contribute to psychological well-being, underscoring the importance of organisational justice and acknowledgement of work effort [18,19,20]. Job satisfaction is closely linked to career-related outcomes, particularly turnover intentions and professional mobility. Dissatisfaction with organisational conditions, rewards, and development opportunities has been consistently associated with increased intention to leave both the current workplace and the nursing profession [21,22,23,24,25,26], with recent evidence highlighting the cumulative impact of sustained workload pressure during and after the COVID-19 pandemic [27]. In the Slovenian healthcare context, previous studies have reported similar patterns, emphasising workload pressure, staffing shortages, leadership quality, and organisational climate as key determinants of nurses’ job satisfaction [5,6,28]. However, existing research has largely focused on hospital-based settings and has often treated job satisfaction as a unidimensional construct. Comprehensive analyses examining multiple dimensions of job satisfaction across different levels of care, while simultaneously accounting for organisational and individual factors, remain limited. The aim of this study was to examine job satisfaction among nursing staff in Slovenian healthcare settings and to analyse the organisational and sociodemographic factors associated with different dimensions of job satisfaction.

## 2. Materials and Methods

### 2.1. Study Design

This research was conducted as a cross-sectional, non-experimental quantitative study. We used an online questionnaire to collect data, which allowed respondents to complete the survey independently and at their convenience. The instrument consisted mainly of structured, closed-ended items.

### 2.2. Population and Sampling Strategy

The study population included nurses and nursing associates working in Slovenian healthcare institutions. A total of 736 nursing professionals working across different levels of healthcare participated in the study. Given the size and dispersion of the target group, we relied on convenience sampling. Although we acknowledge the limitations of this approach, it was the most feasible method for reaching a diverse sample across different clinical environments. Participants were eligible if they held an officially recognised nursing qualification (secondary-level nurse, bachelor-level registered nurse, or a master’s degree in nursing) and if at least 70% of their working time was dedicated to direct patient care. We included individuals from ambulatory care, inpatient hospital care, intensive and emergency units, operating rooms, and labour and delivery units. Participation was voluntary. Participants were grouped according to their job position within the nursing profession. The first group consisted of nurses employed in outpatient settings, the second group included nurses working in hospital wards, and the third group comprised nurses working in emergency departments, intensive care and therapy units, as well as operating and delivery units. Exclusion criteria were defined to ensure consistency in work context: participants working across several units without a dominant setting were excluded, as were those on long-term sick leave or maternity leave. We also excluded nurses whose work was exclusively managerial and did not involve direct care. An a priori power analysis indicated that a minimum of 200 participants per sector (approximately *n* = 600) would be required to achieve at least 0.80 power for the planned multivariate analyses at α = 0.05.

### 2.3. Instrumentation

The questionnaire was organised into three main sections. The first addressed sociodemographic variables (age, gender, education level, years of experience, employment type). The second section captured employment-specific information, including clinical unit, shift pattern, percentage of time spent in direct patient care, and presence of managerial responsibilities. In the third section job satisfaction was assessed using the Job Satisfaction Survey (JSS), a validated instrument developed by Spector that consists of nine subscales assessing different dimensions of job satisfaction. The Job Satisfaction Survey (JSS) was selected because it is a widely validated instrument that allows multidimensional assessment of job satisfaction across nine domains, making it particularly suitable for organisational and healthcare workforce research [29,30]. The JSS consists of 36 items grouped into nine subscales (Pay, Promotion, Supervision, Fringe Benefits, Contingent Rewards, Operating Conditions, Coworkers, Nature of Work, and Communication), with four items per subscale. Responses were recorded on a 6-point Likert-type scale, ranging from 1 (strongly disagree) to 6 (strongly agree), with higher values indicating stronger agreement. Negatively worded items were reverse-scored in accordance with the author’s guidelines prior to analysis, so that higher scores consistently reflected higher job satisfaction. Subscale scores were calculated by summing the four corresponding items, resulting in possible scores ranging from 4 to 24. Total job satisfaction was computed by summing all 36 items, yielding a score range from 36 to 216. Following the absolute scoring approach proposed by Spector, subscale scores were interpreted as follows: scores from 4 to 12 indicate dissatisfaction, 13 to 15 ambivalence, and 16 to 24 satisfaction. For total job satisfaction, scores of 36–108 indicate dissatisfaction, 109–143 ambivalence, and 144–216 satisfaction. The Job Satisfaction Survey (JSS) was administered in Slovenian. The questionnaire was translated and linguistically reviewed to ensure semantic clarity and contextual appropriateness for Slovenian healthcare professionals. Before full deployment, a pilot test was conducted with 15 nurses to evaluate the comprehensibility of the items. Feedback indicated that the wording was generally clear and no substantial revisions were required. Internal consistency was assessed using Cronbach’s alpha, which resulted in α = 0.798, confirming acceptable reliability.

### 2.4. Data Collection Procedure

Data were collected through the 1KA online platform (https://www.1ka.si/ accessed on 27 October 2025). The survey link was distributed via various online channels, including both professional networks and public social media (Facebook, Instagram, Ringaraja.net, and Med.Over.Net). This approach enabled broad dissemination across different segments of the nursing workforce. Before starting the questionnaire, participants viewed an information page explaining the study purpose, confidentiality procedures, data handling, and voluntary nature of participation. Electronic informed consent was required to proceed. No identifying personal data were collected. All responses were anonymised and stored in a password-protected location. Following data collection, the dataset was exported to IBM SPSS Statistics version 27 (IBM Corp., Armonk, NY, USA) for analysis.

### 2.5. Statistical Analysis

The statistical analysis began with descriptive measures (means, medians, standard deviations, interquartile ranges, and frequencies) to summarise the sample characteristics and main study variables. Categorical variables such as place of residence and work schedule were coded into predefined categories prior to statistical analysis. The distribution of continuous variables was assessed using the Kolmogorov–Smirnov test. As normality assumptions were not met for several variables, non-parametric statistical procedures were predominantly applied. For selected demographic variables that approximated a normal distribution (e.g., age groups and years of professional experience), one-way analysis of variance (ANOVA) was additionally performed to examine group differences. Differences in job satisfaction across professional groups and clinical settings were examined using the Kruskal–Wallis test. When statistically significant differences were identified, Dunn’s post hoc test with Bonferroni correction was applied for pairwise comparisons. Associations between job satisfaction dimensions and between job satisfaction and selected demographic or work-related variables were analysed using Spearman’s rank correlation coefficients. To examine factors associated with different dimensions of job satisfaction, multiple linear regression analyses were performed. Each Job Satisfaction Survey (JSS) subscale score was analysed as a dependent variable, while sociodemographic, occupational, and organisational characteristics were entered as independent variables. Regression assumptions were assessed prior to analysis. The regression model was used to assess the relative contribution of each predictor while controlling for the effects of the other variables included in the model. A *p*-value < 0.05 was considered statistically significant throughout the analyses.

## 3. Results

A total of 736 nurses were included in the analysis and categorized according to their job position by level of care: outpatient settings (Group 1, *n* = 220), hospital wards (Group 2, *n* = 240), and emergency departments, intensive care and therapy units, as well as operating and delivery units (Group 3, *n* = 276). No statistically significant differences were observed between groups with respect to age (*p* = 0.124) or total work experience (*p* = 0.173). Similarly, no significant between-group differences were found for place of residence (*p* = 0.111) or work schedule (*p* = 0.239), indicating a largely comparable sociodemographic profile across job positions (Table 1 and Table 2).

Overall, the highest median scores were observed for intrinsic components of work, particularly Nature of Work, Coworkers, and Supervision. In contrast, lower satisfaction levels were reported for Pay, Promotion, and Contingent Rewards, indicating more pronounced dissatisfaction with extrinsic and reward-related aspects. Across most JSS dimensions, nurses working in hospital wards (Group 2) reported the highest median satisfaction scores, whereas Groups 1 and 3 demonstrated lower and largely comparable levels of satisfaction. This pattern was evident for *Pay*, *Promotion*, *Supervision*, *Contingent Rewards*, *Communication*, and *Total Job Satisfaction*. Kruskal–Wallis tests identified statistically significant between-group differences for several JSS dimensions (Table 3). Significant differences were observed for *Pay* (*p* < 0.001), *Promotion* (*p* < 0.001), *Supervision* (*p* < 0.001), *Fringe Benefits* (*p* < 0.001), *Contingent Rewards* (*p* < 0.001), *Communication* (*p* < 0.001), and *Total Job Satisfaction* (*p* < 0.001). No statistically significant differences were identified for *Operating Conditions*, *Coworkers*, or *Nature of Work*.

Post hoc analyses with Bonferroni correction (Table 4) demonstrated consistent patterns across satisfaction domains. For *Pay*, nurses working in hospital wards (Group 2) and high-intensity clinical settings (Group 3) reported significantly higher satisfaction compared with outpatient nurses (Group 1), while no difference was observed between Groups 2 and 3. For *Promotion*, statistically significant differences were observed between all group pairs, with Group 2 reporting the highest satisfaction, followed by Group 3, and the lowest scores observed in Group 1. Satisfaction with *Supervision* was significantly higher in Group 2 compared with both Groups 1 and 3, whereas no difference was observed between Groups 1 and 3. Similar patterns were observed for *Contingent Rewards*, where Group 2 reported significantly higher satisfaction than Groups 1 and 3, while no significant difference was observed between Groups 1 and 3. For *Fringe Benefits*, statistically significant differences were observed between all group pairs, with Group 2 reporting the highest satisfaction and Group 1 the lowest. *Communication* differed significantly between all groups, with the highest scores observed in Group 2 and the lowest in Group 3. *Total Job Satisfaction* was significantly higher in Group 2 compared with Groups 1 and 3, whereas no statistically significant difference was observed between Groups 1 and 3. In most statistically significant comparisons, nurses working in hospital wards (Group 2) demonstrated higher satisfaction scores compared with nurses working in outpatient settings (Group 1) and high-intensity clinical settings (Group 3).

With multiple regression analysis (Table 5) we identified the strongest predictors for each JSS dimension. Negative predictors of Pay satisfaction included higher education level, a higher number of working hours, unclear work tasks, insufficient staffing levels, unfavourable work schedules, and continuous healthcare provision. Positive predictors included higher income, overtime work compensation, and both internal and external job mobility. Promotion satisfaction was negatively associated with higher education, increased working hours, unclear job tasks, unfavourable work schedules, and continuous healthcare provision. Positive predictors included residence in larger settlements, overtime work compensation, and job mobility. Positive predictors of Supervision satisfaction included age, tenure in the current position, financial performance bonus, overtime work compensation, and job mobility. Negative predictors included total work experience, unclear job tasks, indicators of increased workload (Number of working hours, Work schedule, Continuous healthcare provision, Permanent on-call duty), and employer-related characteristics. Lower satisfaction with fringe benefits was associated with lower income, shorter meal breaks, indicators of increased workload, and continuous healthcare provision. Positive predictors included age, higher education level, financial performance bonus, overtime work compensation, and job mobility. Negative predictors of satisfaction with contingent rewards included partnership status, a higher number of working hours, unclear job tasks, compensation for increased workload, job position by level of care and continuous healthcare provision. Positive predictors included higher income, place of residence, employer type, overtime work compensation, and job mobility. Operating conditions were negatively associated with age, partnership status, insufficient staffing levels, shorter meal breaks, utilization of annual leave and continuous healthcare provision. Positive associations were observed for employer type, total work experience and financial performance bonus. Lower satisfaction with coworker relations was associated with higher education level, job position by level of care, partnership status, unclear job tasks, higher number of working hours, utilization of annual leave, and continuous healthcare provision. Positive predictors included higher income, employer type, overtime work compensation, and job mobility. Positive predictors of satisfaction with the nature of work included age, higher education level, residence in larger settlements, overtime work compensation, tenure in current position and compensation for increased workload. Negative predictors included lower income and private employer. Communication was negatively associated with unclear job tasks, lower income, staffing shortages, shorter meal breaks, and continuous healthcare provision. Positive predictors included age, tenure in the current position, employer type, financial performance bonus, and overtime work compensation. Total job satisfaction was negatively associated with unclear job tasks, continuous healthcare provision, employer-related characteristics, shorter meal breaks, higher education level, longer total work experience and absence of compensation for increased workload. Positive predictors included overtime work compensation, age, tenure in the current position, employer type and job mobility.

Spearman correlation analysis (Table 6) revealed multiple statistically significant associations between sociodemographic, occupational, and organizational variables and the JSS subscales, as well as total job satisfaction. Although most correlations were of weak to moderate magnitude, the direction and consistency of associations were largely concordant with the regression findings. Indicators of workload (working hours, unclear job tasks, insufficient staffing levels, limited use of meal breaks and annual leave, and continuous healthcare provision) demonstrated consistent negative correlations with most JSS dimensions and total job satisfaction. In contrast, compensation-related variables, particularly overtime work compensation, showed positive correlations across multiple satisfaction domains. Indicators of job mobility exhibited the strongest correlations with dissatisfaction across all JSS dimensions and total job satisfaction. Overall, the correlation analysis supported the multivariable regression findings, suggesting that workload burden, organizational factors, and job mobility intentions are key determinants of job satisfaction among nurses across different levels of care.

## 4. Discussion

The present study suggests that job satisfaction among nursing staff is more strongly associated with organisational and workload-related factors than with sociodemographic characteristics. In both regression and correlation analyses, organisational indicators such as workload intensity, task clarity, staffing adequacy, and continuous healthcare provision showed consistent associations across several job satisfaction dimensions. Overall, the findings indicate that organisational and workload-related variables demonstrated broader and more consistent associations with job satisfaction than sociodemographic characteristics. While demographic factors showed domain-specific effects, organisational conditions such as task clarity, staffing adequacy, workload intensity, and work organisation were linked to multiple dimensions of job satisfaction, highlighting their central role in shaping nurses’ work experiences. This pattern was observed consistently across both multiple regression and correlation analyses, supporting the internal coherence of the findings. Across analytical approaches, workload-related factors—including long working hours, continuous healthcare provision, unfavourable work schedules, insufficient staffing levels, and limited opportunities for rest—were associated with lower satisfaction across multiple Job Satisfaction Survey (JSS) dimensions and with overall job satisfaction. Among these factors, unclear job task definitions demonstrated particularly strong and consistent associations, affecting nearly all satisfaction domains. These findings are consistent with previous research identifying workload pressure and role ambiguity as important contributors to job dissatisfaction, stress, and burnout among nurses [7,8,9]. Similar associations between workload intensity, staffing adequacy, and job satisfaction have been reported across hospital and high-acuity settings, including intensive care and emergency departments [10,11,12,13]. Studies conducted in home care and inpatient contexts further indicate that insufficient staffing and missed care are linked to both reduced job satisfaction and compromised work experiences [12,14]. Role clarity has been shown to reduce uncertainty and cognitive strain in high-demand work environments, whereas poorly defined responsibilities may intensify perceived stress even in otherwise supportive organisational settings [31,32]. Several organisational characteristics, including employer type, supervision, communication, and staffing adequacy, were associated with satisfaction with supervision, communication, operating conditions, and total job satisfaction. Continuous healthcare provision and unfavourable work schedules were consistently related to lower satisfaction across several domains, suggesting that sustained organisational strain extends beyond workload perception to affect relational and procedural aspects of work. These findings correspond with literature emphasising the importance of leadership behaviours, organisational support, and communication quality in shaping nurses’ job satisfaction [15,16,17]. Recent evidence suggests that planned changes in leadership practices, staffing models, and skill mix may influence nurses’ perceptions of the working environment and job satisfaction, although effects appear to be context-dependent [33,34]. Similar conclusions have been reported in Slovenian studies identifying leadership competencies and organisational climate as relevant determinants of nurses’ job satisfaction [5,6]. A notable finding concerns the role of overtime work compensation. In contrast to most workload indicators, compensated overtime was positively associated with several job satisfaction dimensions, including pay, promotion, supervision, communication, and total job satisfaction. By comparison, compensation for increased workload showed negative associations with selected satisfaction domains, suggesting that different forms of additional work may be perceived differently by nursing staff. These results align with literature emphasising perceived fairness, recognition, and reward structures as important determinants of job satisfaction and well-being [18,19,20,35]. Career-related variables showed domain-specific associations with job satisfaction. Older age and longer tenure in the current position were generally associated with higher satisfaction, particularly with supervision, nature of work, and overall job satisfaction. Indicators of job mobility demonstrated the strongest and most consistent associations across nearly all JSS dimensions. Similar relationships between job dissatisfaction, work environment, and turnover intention have been reported across nursing contexts [21,22,23,24,25,26,36,37]. Evidence from studies conducted during and after the COVID-19 pandemic further highlights the role of sustained work strain in shaping nurses’ intentions to leave the profession [27]. From a practical perspective, the findings underscore the importance of organisational-level interventions targeting workload management, role clarity, leadership quality, and fairness in compensation systems. Such measures may contribute to improved job satisfaction and retention of nursing staff. Future research should employ longitudinal and mixed-methods designs to better assess causal relationships and to examine how organisational interventions, leadership practices, and work environment characteristics influence different dimensions of job satisfaction across healthcare settings. Importantly, these findings have implications beyond the immediate study context. In Slovenia, where nursing workforce shortages and organisational pressures are increasingly pronounced, the results provide empirically grounded evidence to inform workforce planning and organisational reform aimed at improving working conditions and retaining nursing staff. More broadly, the observed pattern—where job satisfaction is driven primarily by organisational and workload-related factors rather than by sociodemographic characteristics—mirrors trends reported internationally, suggesting that the identified mechanisms are likely relevant across diverse healthcare systems. The strong negative association observed between task clarity and communication satisfaction should also be interpreted in the context of variable coding, where higher values indicated less clearly defined job tasks. This finding therefore reflects that poorer role clarity is associated with lower satisfaction with organisational communication. As such, the present findings contribute to the growing international evidence base supporting organisational-level strategies as a central component of efforts to strengthen nursing workforce sustainability and quality of care.

### Study Limitations

This study has several strengths, including a large and diverse sample of nursing staff and the use of the validated Job Satisfaction Survey, which enabled assessment across multiple dimensions of job satisfaction. The inclusion of a broad range of occupational and organisational variables allowed for a comprehensive analysis of factors associated with job satisfaction across healthcare settings. Several limitations should be acknowledged. The cross-sectional design precludes causal inferences, and convenience sampling may limit the generalisability of the findings. Reliance on self-reported data may have introduced response or social desirability bias. As participation was voluntary and conducted via an online survey, some degree of selection bias cannot be excluded, as nurses with particular work experiences or stronger views may have been more likely to participate.

## 5. Conclusions

This study demonstrates that job satisfaction among nursing staff is influenced predominantly by structural and organizational factors rather than by demographic characteristics alone. Although intrinsic aspects of nursing work—such as the perceived meaningfulness of clinical practice and collegial relationships—remain relatively strong, extrinsic factors, including remuneration, opportunities for promotion, and contingent rewards, continue to represent key areas of dissatisfaction. The most prominent negative determinants of job satisfaction were unclear task definitions, continuous work without adequate breaks, excessive workload, insufficient staffing levels, and shortcomings in leadership. These factors consistently affected nearly all dimensions of the Job Satisfaction Survey and emerged as the strongest predictors of overall job satisfaction. Conversely, supportive supervision, higher perceived employer quality, voluntary and appropriately compensated overtime, opportunities for internal or external job mobility, longer work experience, and older age were associated with higher levels of satisfaction. Overall, the findings highlight the need for targeted organizational and structural interventions to improve job satisfaction among nursing staff. Clearer role delineation, adequate staffing, protected rest periods, and strengthened leadership practices appear essential. In addition, fostering career mobility and providing tailored professional development opportunities—particularly for highly educated nurses—may further enhance job satisfaction and staff retention. Addressing these organizational determinants is likely to contribute to a more sustainable nursing workforce, reduce the risk of burnout, and ultimately improve the quality and continuity of patient care.

## Figures and Tables

**Table 1:** Sociodemographic characteristics of the sample by job position and level of care (continuous variables).

Sociodemographic Variables (Linear)	Job Position by Level of Care *	Participants No. (N = 736)	Mean (±Std. Dev.)	*p* Value
Age (Years)	1	220	36.90 ± 5.440	
	2	240	37.71 ± 6.140	0.124
	3	276	37.36 ± 5.870	
Total work experience	1	220	14.81 ± 6.910	
	2	240	15.37 ± 6.430	0.173
	3	276	15.48 ± 6.490	

* 1—outpatient settings, 2—hospital wards, 3—emergency departments, intensive care and therapy units, operating and delivery units.

**Table 2:** Sociodemographic characteristics of the sample by job position and level of care (categorical variables).

Sociodemographic Variables (Categorical)		Job Position by Level of Care—N (%) *			*p* Value
		1	2	3	
Place of residence **	1	121 (55.0%)	112 (56.7%)	160 (58.0%)	0.111
	2	99 (45.0%)	128 (43.3%)	116 (42.0%)	
Work schedule ***	1	44 (20.0%)	64 (26.7%)	64 (23.2%)	0.239
	2	176 (80.0%)	176 (73.3%)	212 (76.8%)	

*—Tableheader: 1—outpatient settings, 2—hospital wards, 3—emergency departments, intensive care and therapy units, operating and delivery units. **—Place of residence: 1—urban settlements; 2—rural settlements. ***—Work schedule: 1—fixed daytime schedule; 2—shift work including night shifts.

**Table 3:** Comparison of Job Satisfaction Survey (JSS) dimensions across job positions and levels of care.

JSS Category	Group * (Job Position by Level of Care)	Participants (n)	Median	*p*-Value **
Pay	1	220	8.50 (5.0–12.75)	**<0.001**
	2	240	11.0 (7.0–15.0)	
	3	276	9.0 (6.0–14.0)	
Promotion	1	220	9.50 (5.5–14.75)	**<0.001**
	2	240	10.50 (9.0–15.0)	
	3	276	10.0 (8.0–14.0)	
Supervision	1	220	15.0 (13.0–19.75)	**<0.001**
	2	240	20.0 (12.0–22.0)	
	3	276	16.0 (14.0–20.0)	
Fringe Benefits	1	220	13.0 (11.0–14.75)	**<0.001**
	2	240	14.0 (12.0–16.0)	
	3	276	13.0 (11.0–15.0)	
Contingent Rewards	1	220	9.50 (6.5–12.0)	**<0.001**
	2	240	12.0 (8.0–14.0)	
	3	276	10.0 (7.0–12.0)	
Operating Conditions	1	220	12.0 (9.25–14.75)	0.456
	2	240	12.0 (10.0–14.0)	
	3	276	12.0 (9.0–14.0)	
Coworkers	1	220	15.50 (12.5–19.5)	0.562
	2	240	16.50 (14.0–18.0)	
	3	276	15.0 (14.0–18.0)	
Nature of Work	1	220	18.50 (17.0–22.0)	0.384
	2	240	20.0 (18.0–21.0)	
	3	276	19.0 (17.0–22.0)	
Communication	1	220	14.0 (11.5–18.0)	**<0.001**
	2	240	17.0 (15.0–20.0)	
	3	276	13.0 (12.0–15.0)	
Total Satisfaction	1	220	117.5 (98.5–135.25)	**<0.001**
	2	240	129.5 (106.0–149.0)	
	3	276	119.0 (106.0–131.0)	

* 1—outpatient settings, 2—hospital wards, 3—emergency departments, intensive care and therapy units, operating and delivery units. ** Kruskal–Wallis test.

**Table 4:** Post hoc pairwise comparisons of Job Satisfaction Survey (JSS) dimensions between job positions and levels of care.

JSS Category	Intergroup Comparison (Job Position by Level of Care) *	*p*-Value **	
		Sig.	Adj. Sig. ***
Pay	1–2	<0.001	**0.040**
	1–3	0.013	**0.029**
	2–3	0.097	0.291
Promotion	1–2	<0.001	**<0.001**
	1–3	0.013	**0.038**
	2–3	<0.001	**0.003**
Supervision	1–2	<0.001	**<0.001**
	1–3	0.256	0.769
	2–3	0.001	**0.004**
Fringe Benefits	1–2	<0.001	**<0.001**
	1–3	0.013	**0.040**
	2–3	0.007	**0.021**
Contingent Rewards	1–2	<0.001	**<0.001**
	1–3	0.259	0.778
	2–3	<0.001	**<0.001**
Operating Conditions	1–2	0.211	0.633
	1–3	0.544	0.633
	2–3	0.483	1.000
Coworkers	1–2	0.729	1.000
	1–3	0.297	0.891
	2–3	0.483	1.000
Nature of Work	1–2	0.167	0.500
	1–3	0.435	1.000
	2–3	0.507	1.000
Communication	1–2	<0.001	**<0.001**
	1–3	0.001	**0.003**
	2–3	<0.001	**<0.001**
Total Satisfaction	1–2	<0.001	**<0.001**
	1–3	0.253	0.760
	2–3	<0.001	**<0.001**

* 1—outpatient settings, 2—hospital wards, 3—emergency departments, intensive care and therapy units, operating and delivery units. ** Pairwise comparisons. *** Significance values adjusted by the Bonferroni correction for multiple test.

**Table 5:** Multiple linear regression analysis of sociodemographic, occupational, and organisational predictors of Job Satisfaction Survey (JSS) dimensions.

**PAY**	**Unstandardized Coefficients** **B**	**Standardized Coefficients** **Beta**	**95.0% Confidence Interval for B**	*p*-Value *
Age	−0.038	−0.065	(−0.236–0.160)	0.708
Education level	−2.426	−0.277	(−3.063–−1.790)	**<0.001**
Housing status	0.154	0.033	(−0.179–0.487)	0.364
Income	1.969	0.256	(1.215–2.722)	**<0.001**
Place of residence	0.039	0.004	(−0.701–0.779)	0.917
Total work experience	0.065	0.117	(−0.121–0.251)	0.493
Tenure in current position	0.022	0.035	(−0.036–0.081)	0.454
Job position by level of care	−0.244	−0.040	(−0.735–0.247)	0.330
Number of working hours	−0.072	−0.269	(−0.093–−0.050)	**<0.001**
Clearly defined job tasks	−0.817	−0.073	(−1.579–−0.055)	**0.036**
Staffing levels according to standards	−0.907	−0.083	(−1.663–−0.152)	**0.019**
Employer	0.182	0.016	(−0.729–1.092)	0.695
Financial Performance bonus	−1.343	−0.117	(−2.217–−0.468)	**0.003**
Compensation for increased workload	−0.201	−0.020	(−0.889–0.487)	0.566
Overtime work compensation	2.096	0.187	(1.366–2.827)	**<0.001**
Use of meal break	−0.835	−0.072	(−1.601–−0.068)	**0.033**
Intention for Job change within the healthcare system	1.366	0.132	(0.631–2.101)	**<0.001**
Intention for Job change outside the healthcare system	1.329	0.132	(0.627–2.031)	**<0.001**
Utilization of annual leave	−0.017	−0.002	(−0.744–0.711)	0.964
Work schedule	−1.829	−0.154	(−2.689–−0.968)	**<0.001**
Continuous healthcare provision	−2.077	−0.174	(−2.952–−1.201)	**<0.001**
Permanent on-call duty	0.102	0.008	(−0.785–0.989)	0.821
**PROMOTION**	**Unstandardized Coefficients** **B**	**Standardized Coefficients** **Beta**	**95.0% Confidence Interval for B**	*p*-Value *
Age	0.013	0.025	(−0.147–0.173)	0.873
Education level	−1.539	−0.197	(−2.054–−1.024)	**<0.001**
Housing status	0.225	0.055	(−0.045–0.494)	0.102
Income	0.231	0.034	(−0.379–0.841)	0.457
Place of residence	0.822	0.092	(0.223–1.421)	**0.007**
Total work experience	0.069	0.139	(−0.082–0.219)	0.370
Tenure in current position	−0.029	−0.050	(−0.076–0.019)	0.238
Job position by level of care	0.092	0.017	(−0.306–0.489)	0.651
Number of working hours	−0.049	−0.206	(−0.066–−0.032)	**<0.001**
Clearly defined job tasks	−1.940	−0.194	(−2.557–−1.323)	**<0.001**
Staffing levels according to standards	0.397	0.041	(−0.214–1.009)	0.203
Employer	−0.825	−0.084	(−1.562–−0.088)	**0.028**
Financial Performance bonus	−1.376	−0.134	(−2.084–−0.668)	**<0.001**
Compensation for increased workload	−0.360	−0.040	(−0.917–0.196)	0.204
Overtime work compensation	2.461	0.246	(1.870–3.053)	**<0.001**
Use of meal break	−0.907	−0.088	(−1.527–−0.287)	**0.004**
Intention for Job change within the healthcare system	0.765	0.083	(0.171–1.360)	**0.012**
Intention for Job change outside the healthcare system	0.934	0.104	(0.366–1.503)	**0.001**
Utilization of annual leave	−0.444	−0.050	(−1.033–0.145)	0.140
Work schedule	−1.696	−0.160	(−2.392–−0.999)	**<0.001**
Continuous healthcare provision	−3.014	−0.284	(−3.723–−2.305)	**<0.001**
Permanent on-call duty	−0.223	−0.019	(−0.941–0.495)	0.543
**SUPERVISION**	**Unstandardized Coefficients** **B**	**Standardized Coefficients** **Beta**	**95.0% Confidence Interval for B**	*p*-Value *
Age	0.369	0.602	(0.175–0.564)	**<0.001**
Education level	1.032	0.113	(0.407–1.657)	**0.001**
Housing status	−0.354	−0.073	(−0.681–−0.027)	**0.034**
Income	−1.216	−0.151	(−1.955–−0.476)	**0.001**
Place of residence	−0.224	−0.021	(−0.950–0.502)	0.546
Total work experience	−0.463	−0.797	(−0.646–−0.281)	**<0.001**
Tenure in current position	0.184	0.274	(0.126–0.241)	**<0.001**
Job position by level of care	−0.430	−0.067	(−0.912–0.052)	0.080
Number of working hours	0.004	0.014	(−0.017–0.025)	0.707
Clearly defined job tasks	−3.630	−0.310	(−4.379–−2.882)	**<0.001**
Staffing levels according to standards	−0.319	−0.028	(−1.060–0.423)	0.399
Employer	−0.926	−0.080	(−1.820–−0.033)	**0.042**
Financial Performance bonus	1.759	0.146	(0.900–2.617)	**<0.001**
Compensation for increased workload	−2.336	−0.220	(−3.011–−1.661)	**<0.001**
Overtime work compensation	2.383	0.203	(1.666–3.100)	**<0.001**
Use of meal break	−0.571	−0.047	(−1.323–0.181)	0.136
Intention for Job change within the healthcare system	2.081	0.192	(1.359–2.802)	**<0.001**
Intention for Job change outside the healthcare system	1.148	0.109	(0.459–1.837)	**0.001**
Utilization of annual leave	−0.636	−0.060	(−1.351–0.078)	0.081
Work schedule	−1.125	−0.091	(−1.969–−0.281)	**0.009**
Continuous healthcare provision	−0.286	−0.023	(−1.146–0.573)	0.513
Permanent on-call duty	−0.549	−0.040	(−1.420–0.322)	0.216
**FRINGE BENEFITS**	**Unstandardized Coefficients** **B**	**Standardized Coefficients** **Beta**	**95.0% Confidence Interval for B**	*p*-Value *
Age	0.200	0.566	(0.082–0.319)	**0.001**
Education level	0.445	0.084	(0.064–0.826)	**0.022**
Housing status	0.102	0.037	(−0.097–0.301)	0.314
Income	−0.534	−0.115	(−0.985–−0.083)	**0.020**
Place of residence	0.113	0.019	(−0.330–0.556)	0.616
Total work experience	−0.166	−0.493	(−0.277–−0.054)	**0.004**
Tenure in current position	0.001	0.003	(−0.034–0.036)	0.944
Job position by level of care	0.216	0.058	(−0.077–0.510)	0.148
Number of working hours	−0.038	−0.233	(−0.050–−0.025)	**<0.001**
Clearly defined job tasks	−1.272	−0.188	(−1.728–−0.815)	**<0.001**
Staffing levels according to standards	0.443	0.067	(−0.009–0.895)	0.055
Employer	0.200	0.030	(−0.345–0.745)	0.471
Financial Performance bonus	0.729	0.105	(0.206–1.253)	**0.006**
Compensation for increased workload	−0.752	−0.123	(−1.163–−0.340)	**<0.001**
Overtime work compensation	0.993	0.146	(0.555–1.430)	**<0.001**
Use of meal break	−1.300	−0.187	(−1.758–−0.841)	**<0.001**
Intention for Job change within the healthcare system	0.908	0.145	(0.468–1.347)	**<0.001**
Intention for Job change outside the healthcare system	0.530	0.087	(0.110–0.950)	**0.013**
Utilization of annual leave	−0.559	−0.092	(−0.995–−0.124)	**0.012**
Work schedule	−0.478	−0.067	(−0.993–0.037)	0.069
Continuous healthcare provision	−1.689	−0.235	(−2.213–−1.165)	**<0.001**
Permanent on-call duty	−1.032	−0.130	(−1.563–−0.501)	**<0.001**
**CONTINGENT REWARDS**	**Unstandardized Coefficients** **B**	**Standardized Coefficients** **Beta**	**95.0% Confidence Interval for B**	*p*-Value *
Age	0.097	0.202	(−0.055–0.248)	0.210
Education level	−0.319	−0.045	(−0.804–0.166)	0.198
Housing status	−0.555	−0.147	(−0.809–−0.302)	**<0.001**
Income	0.794	0.126	(0.220–1.369)	**0.007**
Place of residence	0.594	0.072	(0.030–1.158)	**0.039**
Total work experience	−0.107	−0.236	(−0.249–0.035)	0.139
Tenure in current position	0.057	0.109	(0.013–0.102)	**0.012**
Job position by level of care	−0.569	−0.114	(−0.943–−0.195)	**0.003**
Number of working hours	−0.037	−0.169	(−0.053–−0.020)	**<0.001**
Clearly defined job tasks	−1.577	−0.172	(−2.158–−0.996)	**<0.001**
Staffing levels according to standards	0.161	0.018	(−0.415–0.737)	0.584
Employer	2.225	0.247	(1.531–2.919)	**<0.001**
Financial Performance bonus	0.250	0.027	(−0.417–0.916)	0.462
Compensation for increased workload	−1.051	−0.127	(−1.576–−0.527)	**<0.001**
Overtime work compensation	1.596	0.174	(1.039–2.153)	**<0.001**
Use of meal break	0.078	0.008	(−0.506–0.662)	0.794
Intention for Job change within the healthcare system	1.754	0.207	(1.194–2.314)	**<0.001**
Intention for Job change outside the healthcare system	0.843	0.102	(0.308–1.378)	**0.002**
Utilization of annual leave	−1.491	−0.182	(−2.045–−0.936)	**<0.001**
Work schedule	0.510	0.053	(−0.145–1.166)	0.127
Continuous healthcare provision	−3.196	−0.329	(−3.864–−2.529)	**<0.001**
Permanent on-call duty	0.332	0.031	(−0.345–1.008)	0.336
**OPERATING CONDITIONS**	**Unstandardized Coefficients** **B**	**Standardized Coefficients** **Beta**	**95.0% Confidence Interval for B**	*p*-Value *
Age	−0.217	−0.524	(−0.370–−0.065)	**0.005**
Education level	−0.370	−0.060	(−0.859–0.119)	0.138
Housing status	−0.627	−0.192	(−0.883–−0.371)	**<0.001**
Income	−0.243	−0.044	(−0.821–0.336)	0.411
Place of residence	−0.217	−0.030	(−0.785–0.352)	0.455
Total work experience	0.222	0.565	(0.080–0.365)	**0.002**
Tenure in current position	−0.018	−0.040	(−0.063–0.027)	0.432
Job position by level of care	0.059	0.014	(−0.318–0.436)	0.757
Number of working hours	−0.013	−0.066	(−0.029–0.004)	0.135
Clearly defined job tasks	−0.422	−0.053	(−1.008–0.164)	0.158
Staffing levels according to standards	−1.659	−0.213	(−2.240–−1.079)	**<0.001**
Employer	1.339	0.171	(0.640–2.039)	**<0.001**
Financial Performance bonus	0.681	0.084	(0.009–1.353)	**0.047**
Compensation for increased workload	−0.504	−0.070	(−1.033–0.024)	0.061
Overtime work compensation	−0.051	−0.006	(−0.612–0.510)	0.859
Use of meal break	−1.459	−0.178	(−2.048–−0.870)	**<0.001**
Intention for Job change within the healthcare system	0.047	0.006	(−0.517–0.612)	0.870
Intention for Job change outside the healthcare system	0.264	0.037	(−0.276–0.803)	0.337
Utilization of annual leave	−1.738	−0.244	(−2.297–−1.179)	**<0.001**
Work schedule	0.782	0.093	(0.121–1.443)	**0.020**
Continuous healthcare provision	−1.816	−0.215	(−2.489–−1.143)	**<0.001**
Permanent on-call duty	1.662	0.178	(0.980–2.343)	**<0.001**
**COWORKERS**	**Unstandardized Coefficients** **B**	**Standardized Coefficients** **Beta**	**95.0% Confidence Interval for B**	*p*-Value *
Age	0.081	0.190	(−0.057–0.219)	0.251
Education level	−0.520	−0.081	(−0.965–−0.076)	**0.022**
Housing status	−0.262	−0.078	(−0.494–−0.030)	**0.027**
Income	0.791	0.141	(0.266–1.317)	**0.003**
Place of residence	0.409	0.056	(−0.107–0.926)	0.120
Total work experience	−0.101	−0.249	(−0.231–0.029)	0.128
Tenure in current position	0.027	0.057	(−0.014–0.068)	0.202
Job position by level of care	−0.372	−0.083	(−0.715–−0.029)	**0.033**
Number of working hours	−0.050	−0.257	(−0.065–−0.035)	**<0.001**
Clearly defined job tasks	−1.135	−0.139	(−1.668–−0.603)	**<0.001**
Staffing levels according to standards	−0.479	−0.060	(−1.007–0.048)	0.075
Employer	1.083	0.134	(0.447–1.718)	**0.001**
Financial Performance bonus	0.562	0.067	(−0.048–1.173)	0.071
Compensation for increased workload	0.287	0.039	(−0.193–0.767)	0.242
Overtime work compensation	1.680	0.205	(1.170–2.190)	**<0.001**
Use of meal break	−0.498	−0.059	(−1.033–0.037)	0.068
Intention for Job change within the healthcare system	1.559	0.206	(1.046–2.072)	**<0.001**
Intention for Job change outside the healthcare system	0.671	0.091	(0.181–1.161)	**0.007**
Utilization of annual leave	−1.682	−0.229	(−2.190–−1.174)	**<0.001**
Work schedule	0.594	0.069	(−0.007–1.194)	0.053
Continuous healthcare provision	−0.991	−0.114	(−1.602–−0.380)	**0.002**
Permanent on-call duty	0.368	0.038	(−0.252–0.987)	0.244
**NATURE OF WORK**	**Unstandardized Coefficients** **B**	**Standardized Coefficients** **Beta**	**95.0% Confidence Interval for B**	*p*-Value *
Age	0.185	0.472	(0.046–0.323)	**0.009**
Education level	0.726	0.124	(0.282–1.170)	**0.001**
Housing status	−0.095	−0.031	(−0.328–0.137)	0.420
Income	−1.619	−0.315	(−2.145–−1.094)	**<0.001**
Place of residence	1.111	0.165	(0.595–1.627)	**<0.001**
Total work experience	−0.217	−0.586	(−0.347–−0.087)	**0.001**
Tenure in current position	0.123	0.287	(0.082–0.164)	**<0.001**
Job position by level of care	0.018	0.004	(−0.324–0.361)	0.916
Number of working hours	0.054	0.302	(0.039–0.069)	**<0.001**
Clearly defined job tasks	−0.995	−0.133	(−1.527–−0.463)	**<0.001**
Staffing levels according to standards	0.008	0.001	(−0.519–0.535)	0.977
Employer	−1.004	−0.136	(−1.639–−0.369)	**0.002**
Financial Performance bonus	−0.285	−0.037	(−0.895–0.325)	0.359
Compensation for increased workload	0.798	0.118	(0.319–1.278)	**0.001**
Overtime work compensation	1.452	0.194	(0.943–1.962)	**<0.001**
Use of meal break	−0.372	−0.048	(−0.906–0.162)	0.172
Intention for Job change within the healthcare system	1.140	0.165	(0.628–1.653)	**<0.001**
Intention for Job change outside the healthcare system	1.577	0.234	(1.087–2.066)	**<0.001**
Utilization of annual leave	0.834	0.124	(0.326–1.341)	**0.001**
Work schedule	0.868	0.110	(0.268–1.468)	**0.005**
Continuous healthcare provision	0.902	0.113	(0.291–1.512)	**0.004**
Permanent on-call duty	0.616	0.070	(−0.002–1.235)	0.051
**COMMUNICATION**	**Unstandardized Coefficients** **B**	**Standardized Coefficients** **Beta**	**95.0% Confidence Interval for B**	*p*-Value *
Age	0.539	1.094	(0.392–0.687)	**<0.001**
Education level	0.156	0.021	(−0.318–0.630)	0.519
Housing status	−0.181	−0.046	(−0.428–0.067)	0.153
Income	−1.703	−0.263	(−2.265–−1.142)	**<0.001**
Place of residence	−0.045	−0.005	(−0.596–0.506)	0.873
Total work experience	−0.544	−1.165	(−0.683–−0.406)	**<0.001**
Tenure in current position	0.110	0.205	(0.067–0.154)	**<0.001**
Job position by level of care	−0.132	−0.026	(−0.498–0.234)	0.478
Number of working hours	−0.013	−0.056	(−0.029–0.003)	0.120
Clearly defined job tasks	−3.690	−0.392	(−4.258–−3.123)	**<0.001**
Staffing levels according to standards	−1.056	0.114	(–1.619–−0.494)	**<0.001**
Employer	2.478	0.267	(1.800–3.156)	**<0.001**
Financial Performance bonus	1.227	0.127	(0.576–1.879)	**<0.001**
Compensation for increased workload	−0.671	−0.079	(−1.183–−0.159)	**0.010**
Overtime work compensation	0.981	0.104	(0.437–1.525)	**<0.001**
Use of meal break	−0.765	−0.079	(−1.336–−0.194)	**0.009**
Intention for Job change within the healthcare system	1.793	0.206	(1.246–2.340)	**<0.001**
Intention for Job change outside the healthcare system	0.416	0.049	(−0.107–0.938)	0.119
Utilization of annual leave	−0.405	−0.048	(−0.947–0.137)	0.143
Work schedule	−0.549	−0.055	(−1.190–0.092)	0.093
Continuous healthcare provision	−1.970	−0.197	(−2.623–−1.318)	**<0.001**
Permanent on-call duty	0.464	0.042	(−0.196–1.125)	0.168
**TOTAL SATISFACTION**	**Unstandardized Coefficients** **B**	**Standardized Coefficients** **Beta**	**95.0% Confidence Interval for B**	*p*-Value *
Age	1.229	0.408	(0.353–2.104)	**0.006**
Education level	−2.815	−0.063	(−5.627–−0.003)	0.050
Housing status	−1.594	−0.067	(−3.065–−0.123)	**0.034**
Income	−1.529	−0.039	(−4.858–1.799)	0.367
Place of residence	2.603	0.050	(−0.666–5.872)	0.118
Total work experience	−1.242	−0.435	(−2.064–−0.420)	**0.003**
Tenure in current position	0.478	0.145	(0.219–0.737)	**<0.001**
Job position by level of care	−1.361	−0.043	(−3.530–0.807)	0.218
Number of working hours	−0.212	−0.155	(−0.307–−0.118)	**<0.001**
Clearly defined job tasks	−15.478	−0.269	(−18.846–−12.111)	**<0.001**
Staffing levels according to standards	−1.300	−0.023	(−4.637–2.038)	0.445
Employer	4.752	0.084	(0.729–8.774)	**0.021**
Financial Performance bonus	2.205	0.037	(−1.659–6.069)	0.263
Compensation for increased workload	−4.790	−0.092	(−7.829–−1.752)	**0.002**
Overtime work compensation	13.592	0.236	(10.364–16.820)	**<0.001**
Use of meal break	−6.629	−0.112	(−10.014–−3.244)	**<0.001**
Intention for Job change within the healthcare system	11.413	0.214	(8.167–14.660)	**<0.001**
Intention for Job change outside the healthcare system	7.712	0.149	(4.610–10.813)	**<0.001**
Utilization of annual leave	−6.138	−0.119	(−9.353–−2.923)	**<0.001**
Work schedule	−2.922	−0.048	(−6.723–0.878)	0.132
Continuous healthcare provision	−14.138	−0.231	(−18.006–−10.270)	**<0.001**
Permanent on-call duty	1.740	0.026	(−2.180–5.660)	0.384

*—Statistically significant *p*-values are highlighted in bold.

**Table 6:** Spearman correlation coefficients between sociodemographic, occupational, and organisational variables and Job Satisfaction Survey (JSS) dimensions.

JSS—Spearman’s Rho		PAY	PROMOTION	SUPERVISION	FRINGE BENEFITS	CONTINGENT REWARDS	OPERATING CONDITIONS	COWORKERS	NATURE OF WORK	COMMUNICA -TION	TOTAL SATISFACTION
Age	Corr. Coef.	0.158	0.233	0.102	0.111	0.111	0.059	0.018	0.235	0.070	0.199
	*p*-value	0.000	0.000	0.006	0.003	0.002	0.109	0.635	0.000	0.058	0.000
Education level	Corr. Coef.	−0.136	−0.083	0.081	0.137	0.034	−0.090	−0.045	0.080	−0.042	0.017
	*p*-value	0.000	0.025	0.028	0.000	0.355	0.015	0.218	0.030	0.255	0.653
Housing status	Corr. Coef.	0.046	0.018	−0.072	0.015	−0.038	−0.081	0.037	−0.119	−0.001	−0.057
	*p*-value	0.215	0.622	0.051	0.691	0.303	0.028	0.319	0.001	0.976	0.123
Income	Corr. Coef.	0.048	−0.027	0.015	−0.019	0.095	−0.058	−0.018	0.009	−0.131	0.026
	*p*-value	0.193	0.471	0.680	0.600	0.010	0.113	0.628	0.805	0.000	0.478
Place of residence	Corr. Coef.	0.012	0.141	0.017	0.088	0.051	0.070	0.070	0.171	−0.025	0.090
	*p*-value	0.738	0.000	0.651	0.017	0.167	0.056	0.056	0.000	0.493	0.014
Total work experience	Corr. Coef.	0.152	0.249	0.068	0.077	0.089	0.060	−0.003	0.214	0.037	0.175
	*p*-value	0.000	0.000	0.065	0.037	0.015	0.103	0.925	0.000	0.322	0.000
Tenure in current position	Corr. Coef.	0.143	0.114	0.212	0.048	0.177	0.033	0.041	0.190	0.050	0.152
	*p*-value	0.000	0.002	0.000	0.197	0.000	0.365	0.265	0.000	0.177	0.000
Job position by level of care	Corr. Coef.	0.079	0.073	0.027	0.075	0.023	−0.018	−0.039	0.025	−0.154	0.023
	*p*-value	0.031	0.047	0.470	0.041	0.540	0.619	0.288	0.506	0.000	0.535
Number of working hours	Corr. Coef.	−0.262	−0.240	−0.167	−0.291	−0.157	−0.124	−0.410	0.041	−0.222	−0.281
	*p*-value	0.000	0.000	0.000	0.000	0.000	0.001	0.000	0.267	0.000	0.000
Clearly defined job tasks	Corr. Coef.	−0.279	−0.387	−0.386	−0.293	−0.307	−0.185	−0.292	−0.153	−0.430	−0.472
	*p*-value	0.000	0.000	0.000	0.000	0.000	0.000	0.000	0.000	0.000	0.000
Staffing levels according to standards	Corr. Coef.	−0.193	−0.085	−0.243	−0.145	−0.161	−0.222	−0.258	−0.120	−0.111	−0.257
	*p*-value	0.000	0.021	0.000	0.000	0.000	0.000	0.000	0.001	0.003	0.000
Employer	Corr. Coef.	−0.047	−0.225	−0.017	−0.014	0.020	0.011	0.127	−0.140	0.210	−0.064
	*p*-value	0.203	0.000	0.639	0.703	0.580	0.770	0.001	0.000	0.000	0.082
Financial Performance bonus	Corr. Coef.	−0.260	−0.322	−0.072	−0.098	−0.101	−0.063	0.009	−0.081	−0.017	−0.182
	*p*-value	0.000	0.000	0.050	0.008	0.006	0.088	0.814	0.028	0.653	0.000
Compensation for increased workload	Corr. Coef.	−0.115	−0.165	−0.238	−0.194	−0.149	−0.066	0.034	0.065	−0.134	−0.141
	*p*-value	0.002	0.000	0.000	0.000	0.000	0.074	0.361	0.079	0.000	0.000
Overtime work compensation	Corr. Coef.	0.120	0.171	0.185	0.127	0.165	0.054	0.250	0.243	0.112	0.220
	*p*-value	0.001	0.000	0.000	0.001	0.000	0.141	0.000	0.000	0.002	0.000
Use of meal break	Corr. Coef.	−0.153	−0.147	−0.096	−0.175	−0.006	−0.172	−0.091	−0.067	−0.160	−0.187
	*p*-value	0.000	0.000	0.009	0.000	0.865	0.000	0.014	0.068	0.000	0.000
Intention for Job change within the healthcare system	Corr. Coef.	0.267	0.243	0.352	0.291	0.399	0.160	0.387	0.172	0.325	0.444
	*p*-value	0.000	0.000	0.000	0.000	0.000	0.000	0.000	0.000	0.000	0.000
Intention for Job change outside the healthcare system	Corr. Coef.	0.330	0.327	0.241	0.281	0.297	0.133	0.302	0.220	0.220	0.402
	*p*-value	0.000	0.000	0.000	0.000	0.000	0.000	0.000	0.000	0.000	0.000
Utilization of annual leave	Corr. Coef.	−0.119	−0.170	−0.270	−0.248	−0.340	−0.210	−0.377	−0.026	−0.218	−0.325
	*p*-value	0.001	0.000	0.000	0.000	0.000	0.000	0.000	0.484	0.000	0.000
Work schedule	Corr. Coef.	−0.089	−0.141	−0.223	−0.173	−0.097	−0.003	−0.028	0.100	−0.202	−0.144
	*p*-value	0.016	0.000	0.000	0.000	0.009	0.944	0.453	0.007	0.000	0.000
Continuous healthcare provision	Corr. Coef.	−0.155	−0.276	−0.042	−0.151	−0.262	−0.098	0.019	0.013	0.000	−0.192
	*p*-value	0.000	0.000	0.254	0.000	0.000	0.008	0.609	0.722	0.991	0.000
Permanent on-call duty	Corr. Coef.	0.037	0.015	−0.094	−0.154	0.012	0.167	0.038	0.026	0.100	0.023
	*p*-value	0.317	0.688	0.010	0.000	0.736	0.000	0.309	0.482	0.006	0.526

## Data Availability

The data presented in this study are available from the corresponding author upon reasonable request.

## Public Involvement Statement

Patients and the public were not involved in the design, conduct, analysis, or dissemination of this study. The research focused exclusively on nursing staff and their job satisfaction within Slovenian healthcare settings, using standardized self-report questionnaires.

## Guidelines and Standards Statement

This cross-sectional study was conducted in accordance with the Declaration of Helsinki and reported in line with the Strengthening the Reporting of Observational Studies in Epidemiology (STROBE) guidelines.

## Use of Artificial Intelligence

The authors disclose that artificial intelligence–based tools were used (ChatGPT, OpenAI, San Francisco, CA, USA; model version GPT-5.3) solely for language editing and proofreading of the manuscript, as English is not the authors’ native language. The use of these tools was limited to improving clarity, grammar, and stylistic consistency. All scientific content, data analysis, interpretation of results, and conclusions were developed by the authors, who take full responsibility for the integrity of the work.
